# Supplementary material for: Vertical Slot Fishways: Incremental Knowledge to Define the Best Solution
Source: Biology (Basel). 2023 Nov 15;12(11):1431. doi: 10.3390/biology12111431 (PMC10669019; doi:10.3390/biology12111431)
Supplement: Supplementary file 1 [file biology-12-01431-s001.zip › biology-2643880-supplementary.pdf]

Supplementary material

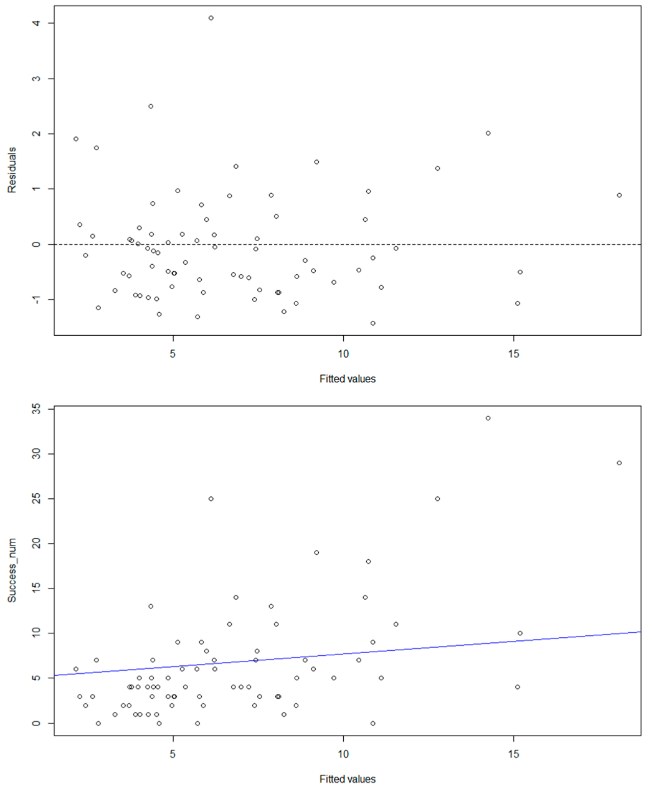

Figure S1. – Graphical validation for the model concerning the first question "What variables affect fish passage during experimental fishway studies?". Pearson residuals versus fitted values on the left and observed data versus fitted values on the right.

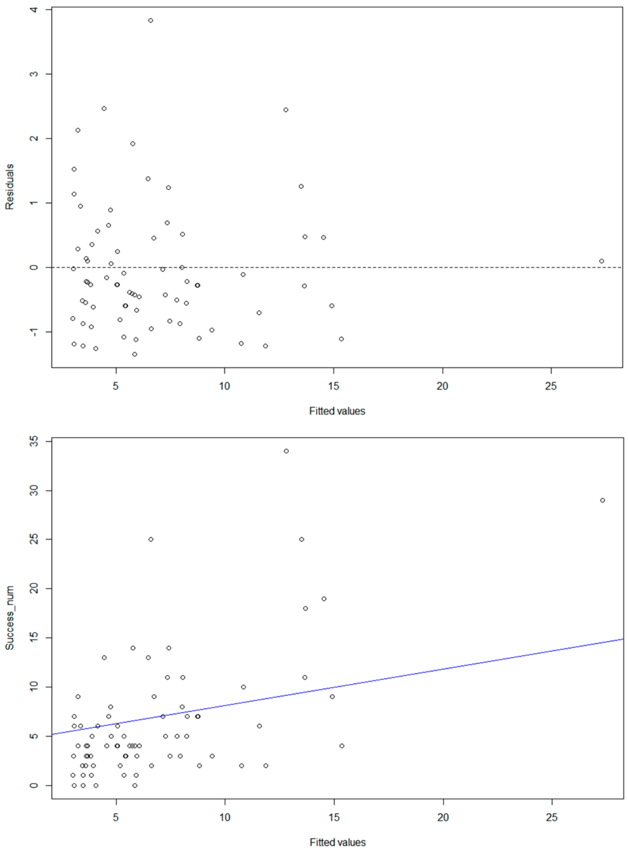

Figure S2. – Graphical validation for the model concerning the second question "What is the best VSF configuration?". Pearson residuals versus fitted values on the left and observed data versus fitted values on the right.

Table S1. – Details of the variables excluded from the modelling procedures.

Type of Variable	Variable	Acronym	Description	Unit/levels
Factor	Year	Time_year	Identification of the year in which the experiment took place	2015 and 2016
Factor	Month	Time_month	Identification of the month in which the experiment took place	May, June, July, September and October
Continuous	Discharge in the Fishway	Discharge	Discharge used during each experiment	l s^{-1}
Continuous	Average body mass of fish school	Mass_avg	Average body mass considering the five fish present in each fish school	g

Type of Variable	Variable	Acronym	Description	Unit/levels
Continuous	Standard Deviation of body mass of fish school	Mass_std	Standard deviation of body mass considering the five fish present in each fish school	g
Continuous	Standard deviation length of fish school	Length_std	Standard Deviation of the total length considering the five fish present in each fish school	cm
Continuous	Water temperature in the acclimation tanks	Temp_aclim	Water temperature in the acclimation tanks measured before the experiment	° C
Continuous	Water pH in the fishway	pH_PPP	Water pH in the fishway	
